# Bridging the Gaps in Dementia Care: A Call for Integrated Comorbidity Management

**DOI:** 10.1002/hsr2.70788

**Published:** 2025-05-09

**Authors:** Jonathan J. O. Canete

**Affiliations:** ^1^ Department of Innovation and Sustainability De La Salle University Manila Philippines

## Transparency Statement

The lead author affirms that this manuscript is an honest, accurate, and transparent account of the study being reported; that no important aspects of the study have been omitted; and that any discrepancies from the study as originally planned (if applicable) have been explained. All authors have approved the final manuscript and agree to be accountable for all aspects of the work. The authors declare that the study was conducted in accordance with the ethical principles outlined in the Declaration of Helsinki and that all necessary institutional approvals and informed consent procedures were observed.

Dear Editor,

This is in recognition for the recently published research article, *“*Thinking About It All Together: A Descriptive Analysis to Understand Comorbidities in People Living With Dementia*”* by Zhang et al. This study presents an important and timely investigation into the prevalence and patterns of comorbidities among individuals with dementia, shedding light on the complexities of providing person‐centered care to this vulnerable population.

The authors' use of the *All of Us Research Program* data set to analyze comorbidity trends provides valuable insights into the multifaceted nature of dementia care. The study effectively underscores the high prevalence of comorbid conditions among individuals with dementia, with 87% of the study population experiencing at least one additional chronic illness. The findings regarding the most common comorbidities, such as diabetes (67.82%), renal disease (40.24%), and chronic pulmonary disease (39.85%), provide compelling evidence for the necessity of an integrated care approach that considers both dementia and co‐occurring conditions [1].

One of the study's strengths is its use of a large‐scale, diverse data set, which allows for a robust assessment of comorbidity patterns. By employing statistical tools such as UpSet Plots, the study presents a clear visualization of the intricate relationships between multiple chronic conditions in dementia patients. Such a methodological approach enhances the field's understanding of the disease trajectory [2] in persons living with dementia and has important implications for improving care coordination.

However, while the study successfully highlights the prevalence of comorbidities, it also raises significant concerns about the existing healthcare framework for dementia patients. The findings suggest that a substantial proportion of individuals with dementia are at risk of experiencing fragmented care due to the complex interplay of multiple chronic conditions. This raises important questions about whether current healthcare models are adequately equipped to manage these overlapping health burdens in a way that is truly person‐centered and holistic.

An important consideration that the authors could further explore in future research is the impact of these comorbidities on health outcomes such as hospitalization rates, functional decline, and mortality. Additionally, understanding the influence of social determinants of health on comorbidity patterns could provide deeper insights into disparities in dementia care. The study briefly touches on the association between race and comorbidity prevalence, but further research into socioeconomic factors and healthcare access barriers could enhance the depth of this analysis.

Moreover, the study's findings emphasize the need for a paradigm shift in dementia care. The current healthcare approach often prioritizes the management of individual diseases rather than addressing the patient's overall well‐being in an integrated manner. A move toward comprehensive, interdisciplinary care models that incorporate geriatrics, neurology, cardiology, and palliative care could greatly benefit individuals with dementia and their caregivers. In this regard, the authors' findings provide strong support for initiatives such as the *Guiding an Improved Dementia Experience (GUIDE) Model* proposed by the Centers for Medicare and Medicaid Services, which aims to enhance coordinated dementia care.

It is also worth noting that while the study's reliance on electronic health records (EHRs) provides valuable data, it may also introduce limitations in terms of missing or inconsistent information. Given the retrospective and cross‐sectional nature of the study, a longitudinal approach could further strengthen the understanding of how comorbidity patterns evolve over time and their implications for disease progression.

In general, Zhang et al. have made a significant contribution to dementia research by providing an in‐depth descriptive analysis of comorbidity profiles in this population. Their findings highlight the urgent need for a more integrated and person‐centered approach to dementia care. Future research should build upon these findings by exploring the longitudinal impact of comorbidities, addressing disparities in healthcare access, and identifying strategies to improve comprehensive care models for individuals with dementia. I commend the authors for their valuable work and look forward to further discussions on how these insights can be translated into practice.

## Author Contributions

**Jonathan J. O. Canete:** conceptualization, writing – original draft, writing – review and editing.

## Conflicts of Interest

The author declares no conflicts of interest.

## Data Availability

The data that support the findings of this study are openly available in Human Science Reports at https://onlinelibrary.wiley.com/doi/epdf/10.1002/hsr2.70449.
